# Supplementary material for: The CRTC-1 transcriptional domain is required for COMPASS complex-mediated longevity in C. elegans
Source: Nat Aging. 2023 Nov 9;3(11):1358–71. doi: 10.1038/s43587-023-00517-8 (PMC10645585; doi:10.1038/s43587-023-00517-8)
Supplement: Supplementary file 1 — Supplementary Fig. 1. [file 43587_2023_517_MOESM1_ESM.pdf]

nature aging

Article

https://doi.org/10.1038/s43587-023-00517-8

# The CRTC-1 transcriptional domain is required for COMPASS complex-mediated longevity in *C. elegans*

In the format provided by the authors and unedited

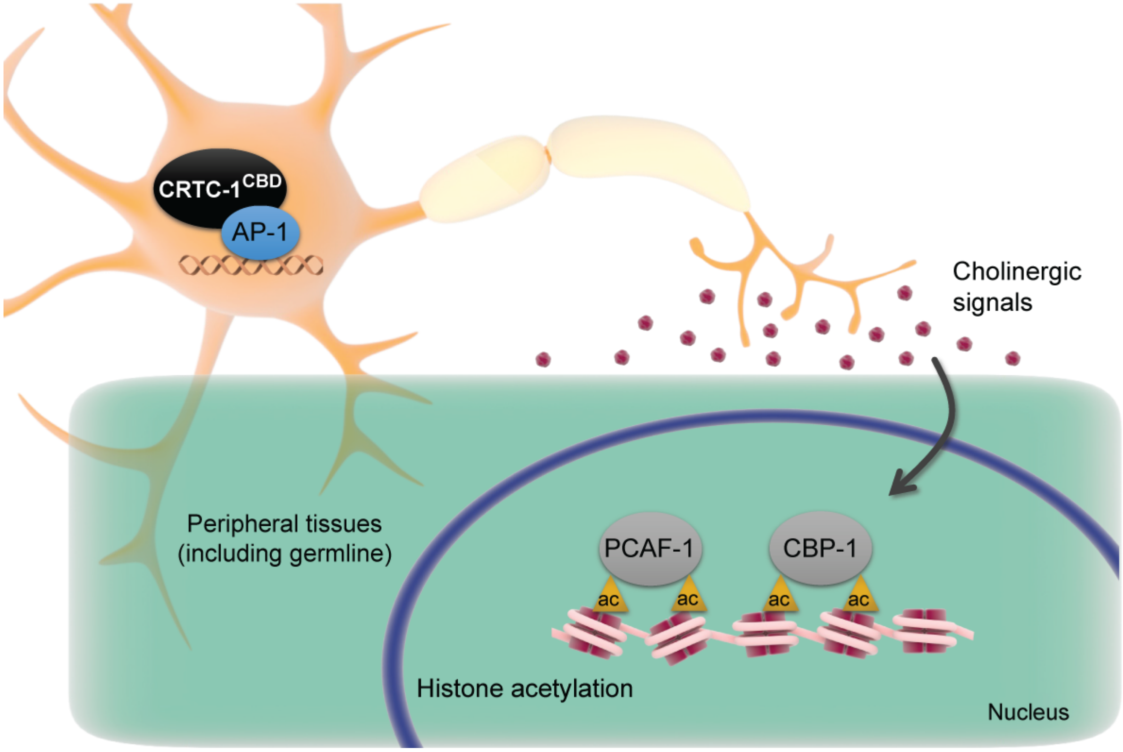


**Supplementary Fig. 1. Model depicting CRTC-1 requirement for COMPASS-dependent longevity.** Lifespan extension induced by H3K4me3 methyltransferase deficiency requires a specific function (co-transcriptional regulation) of CRTC-1 driven by its CREB-binding domain (CBD), which in turn triggers a neuron-to-periphery cholinergic signal via the transcription factor AP-1. This signal then leads to activation of histone acetylation and gene expression.
