# Supplementary material for: The CRTC-1 transcriptional domain is required for COMPASS complex-mediated longevity in C. elegans
Source: Nat Aging. 2023 Nov 9;3(11):1358–71. doi: 10.1038/s43587-023-00517-8 (PMC10645585; doi:10.1038/s43587-023-00517-8)
Supplement: Supplementary file 2 — Reporting Summary [file 43587_2023_517_MOESM2_ESM.pdf]

nature portfolio

Corresponding author(s): William B. Mair

Last updated by author(s): Jul 29, 2023

Reporting Summary

Nature Portfolio wishes to improve the reproducibility of the work that we publish. This form provides structure for consistency and transparency in reporting. For further information on Nature Portfolio policies, see our Editorial Policies and the Editorial Policy Checklist.

Statistics

For all statistical analyses, confirm that the following items are present in the figure legend, table legend, main text, or Methods section.

n/a	Confirmed	
☐	☒	The exact sample size (*n*) for each experimental group/condition, given as a discrete number and unit of measurement
☐	☒	A statement on whether measurements were taken from distinct samples or whether the same sample was measured repeatedly
☐	☒	The statistical test(s) used AND whether they are one- or two-sided<br>*Only common tests should be described solely by name; describe more complex techniques in the Methods section.*
☒	☐	A description of all covariates tested
☐	☒	A description of any assumptions or corrections, such as tests of normality and adjustment for multiple comparisons
☐	☒	A full description of the statistical parameters including central tendency (e.g. means) or other basic estimates (e.g. regression coefficient) AND variation (e.g. standard deviation) or associated estimates of uncertainty (e.g. confidence intervals)
☐	☒	For null hypothesis testing, the test statistic (e.g. *F*, *t*, *r*) with confidence intervals, effect sizes, degrees of freedom and *P* value noted<br>*Give P values as exact values whenever suitable.*
☒	☐	For Bayesian analysis, information on the choice of priors and Markov chain Monte Carlo settings
☒	☐	For hierarchical and complex designs, identification of the appropriate level for tests and full reporting of outcomes
☒	☐	Estimates of effect sizes (e.g. Cohen's *d*, Pearson's *r*), indicating how they were calculated

Our web collection on statistics for biologists contains articles on many of the points above.

Software and code

Policy information about availability of computer code

Data collection	Zen blue edition (version 3.0)<br>SoftWoRX (version 3.3.6)<br>MassHunter GC/MS Acquisition (version 10.0.368)
Data analysis	RNA-seq data analysis was performed using<br>R (v3.6.2) using the packages:<br>FastQC (v0.11.9)<br>STAR58 (v2.7.10a)<br>Salmon (v1.1.0)<br>Bioconductor (v3.10)<br>tximport (v1.12.3)<br>DESeq261 (v1.24.0)<br>clusterProfiler (v3.14.3)<br>DEGreport (v1.20.0)<br>Other analysis tools used include:<br>Fiji (version 2.0.0)<br>Prism (version 9)<br>Enrichment Analysis WormBase (version WS280)<br>Perseus software (version 1.5.1.6)<br>STRING (version 11.0)

oPOSSUM (version 3.0)
MaxQuant software (version 1.5.2.8)

For manuscripts utilizing custom algorithms or software that are central to the research but not yet described in published literature, software must be made available to editors and reviewers. We strongly encourage code deposition in a community repository (e.g. GitHub). See the Nature Portfolio guidelines for submitting code & software for further information.

Data

Policy information about availability of data

All manuscripts must include a data availability statement. This statement should provide the following information, where applicable:
- Accession codes, unique identifiers, or web links for publicly available datasets
- A description of any restrictions on data availability
- For clinical datasets or third party data, please ensure that the statement adheres to our policy

All raw and processed sequencing data for RNA-seq libraries can be found under NCBI Gene Expression Omnibus (GEO) ID GSE239572. All raw and processed data for proteomics can be found under ProteomeXchange ID PXD045747. Source data are provided with this article.

Human research participants

Policy information about studies involving human research participants and Sex and Gender in Research.

Reporting on sex and gender	No human research participants were included in this study.
Population characteristics	No human research participants were included in this study.
Recruitment	No human research participants were included in this study.
Ethics oversight	No human research participants were included in this study.

Note that full information on the approval of the study protocol must also be provided in the manuscript.

Field-specific reporting

Please select the one below that is the best fit for your research. If you are not sure, read the appropriate sections before making your selection.

☒ Life sciences ☐ Behavioural & social sciences ☐ Ecological, evolutionary & environmental sciences

For a reference copy of the document with all sections, see nature.com/documents/nr-reporting-summary-flat.pdf

Life sciences study design

All studies must disclose on these points even when the disclosure is negative.

Sample size	No statistical methods were used to select sample size. Each type of experiment requires different number of individuals. For example, 100 animals for lifespan and more than 400,000 animals for immunoprecipitation experiments. We always maintain a high sample size as possible according to our standard protocols.
Data exclusions	No data were excluded from the analyses.
Replication	All experiments have at least 3 repeats in completely independent and successful experiments.
Randomization	Worms were randomly distributed into treatments and controls groups. Lifespan assays and fluorescent imaging was performed randomized. RNA-seq, lipidomic and IP samples were performed randomized. In all experiments, the covariates were the same for all conditions and animals.
Blinding	Experiments were not blinded. However, samples and data were collected in batches for at least three completely independent biological replicates to ensure that the results are consistent and replicable.

Reporting for specific materials, systems and methods

We require information from authors about some types of materials, experimental systems and methods used in many studies. Here, indicate whether each material, system or method listed is relevant to your study. If you are not sure if a list item applies to your research, read the appropriate section before selecting a response.

Materials & experimental systems

n/a	Involved in the study
☐	☒ Antibodies
☒	☐ Eukaryotic cell lines
☒	☐ Palaeontology and archaeology
☐	☒ Animals and other organisms
☒	☐ Clinical data
☒	☐ Dual use research of concern

Methods

n/a	Involved in the study
☒	☐ ChIP-seq
☒	☐ Flow cytometry
☒	☐ MRI-based neuroimaging

Antibodies

Antibodies used	α-H3K4me3 (MAB Institute Inc Wako 305-34819), α-H3K9ac (Abcam ab10812), α-mouse Cy-3 (Jackson ImmunoResearch Laboratories 115-165-003), α-rabbit Alexa 488 (Jackson ImmunoResearch Laboratories 111-545-003), α-H3K4me3 (Abcam ab8580), α-H3K18ac (Sigma-Aldrich 07-354), rabbit α-H3K27ac (Abcam ab4729), rabbit α-H3 (Cell Signaling 9715), and α-rabbit IgG HRP-linked (Cell Signaling 7074).
Validation	All the antibodies used in this work are commercially available and have been published/cited:<br>https://www.citeab.com/antibodies/search?q=Abcam+ab10812<br>https://www.citeab.com/antibodies/search?q=Abcam+ab8580<br>https://www.citeab.com/antibodies/search?q=Sigma-Aldrich+07-354<br>https://www.citeab.com/antibodies/search?q=Abcam+ab4729<br>https://www.citeab.com/antibodies/search?q=Cell+Signaling+9715<br>https://www.citeab.com/antibodies/search?q=Cell+Signaling+7074<br>https://www.citeab.com/antibodies/2036026-115-165-003-cy3-affinipure-goat-anti-mouse-igg-h-l?des=c68c800c133c6a0d<br>https://www.citeab.com/antibodies/2034512-111-545-003-alexa-fluor-488-affinipure-goat-anti-rab?des=8b93fd547c9aae73<br>https://compbio.med.harvard.edu/antibodies/antibodies/219 & https://pubmed.ncbi.nlm.nih.gov/18227620/

Animals and other research organisms

Policy information about studies involving animals; ARRIVE guidelines recommended for reporting animal research, and Sex and Gender in Research

Laboratory animals	Caenorhabditis elegans hermaphrodites at the age of 1 day of adulthood were used for all experiments except for lifespan assays, where worms were monitored throughout their lifespan.
Wild animals	The study did not involve wild animals.
Reporting on sex	All animals were hermaphrodite.
Field-collected samples	The study did not involve samples collected from the field.
Ethics oversight	Harvard University supervises and approves the study with C. elegans.

Note that full information on the approval of the study protocol must also be provided in the manuscript.
