# Supplementary figures and images for: The CRTC-1 transcriptional domain is required for COMPASS complex-mediated longevity in C. elegans
Source: Nat Aging. 2023 Nov 9;3(11):1358–71. doi: 10.1038/s43587-023-00517-8 (PMC10645585; doi:10.1038/s43587-023-00517-8)

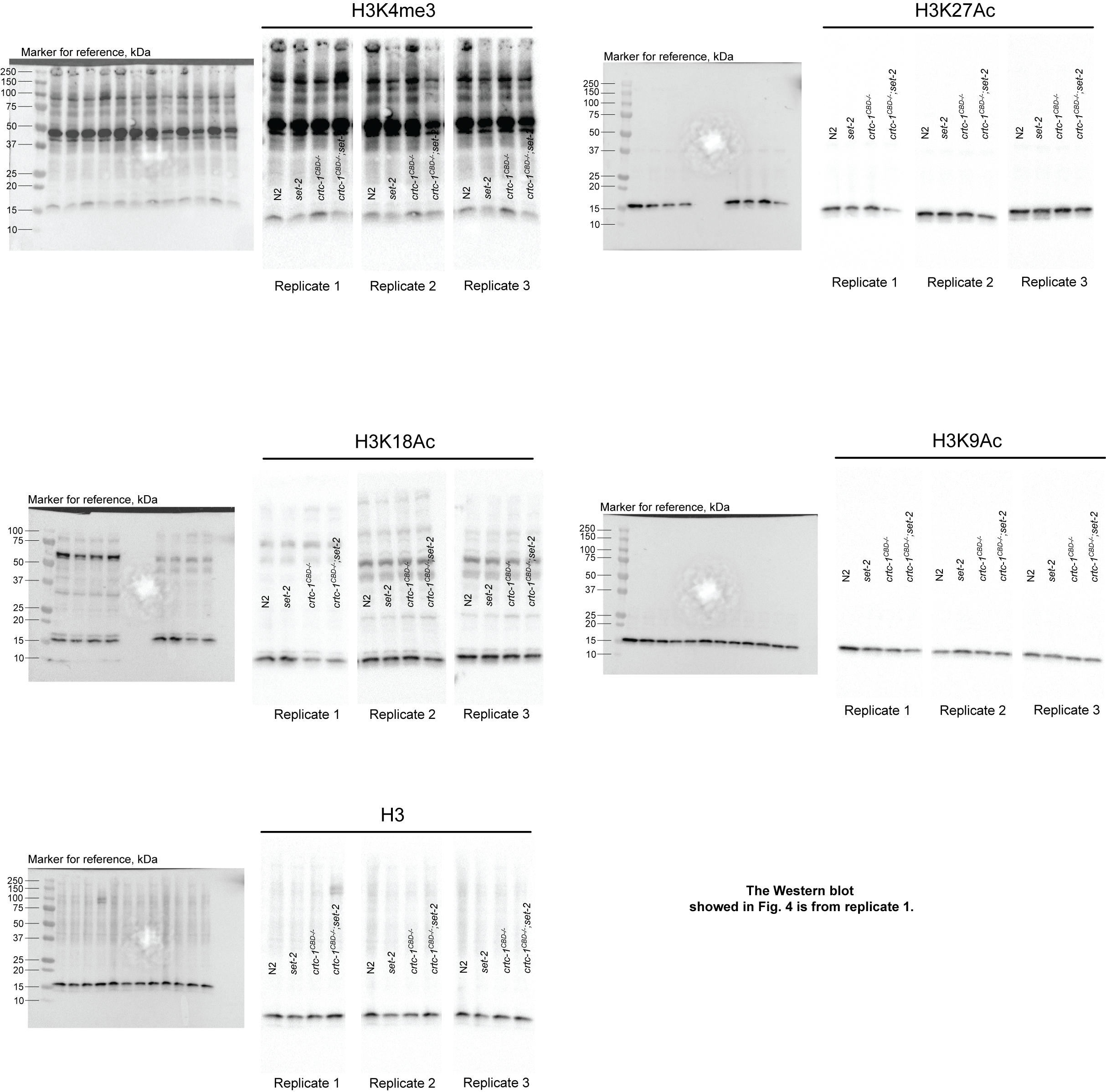

Supplement: Supplementary file 12 — Unprocessed western blots. [file 43587_2023_517_MOESM12_ESM.tif]

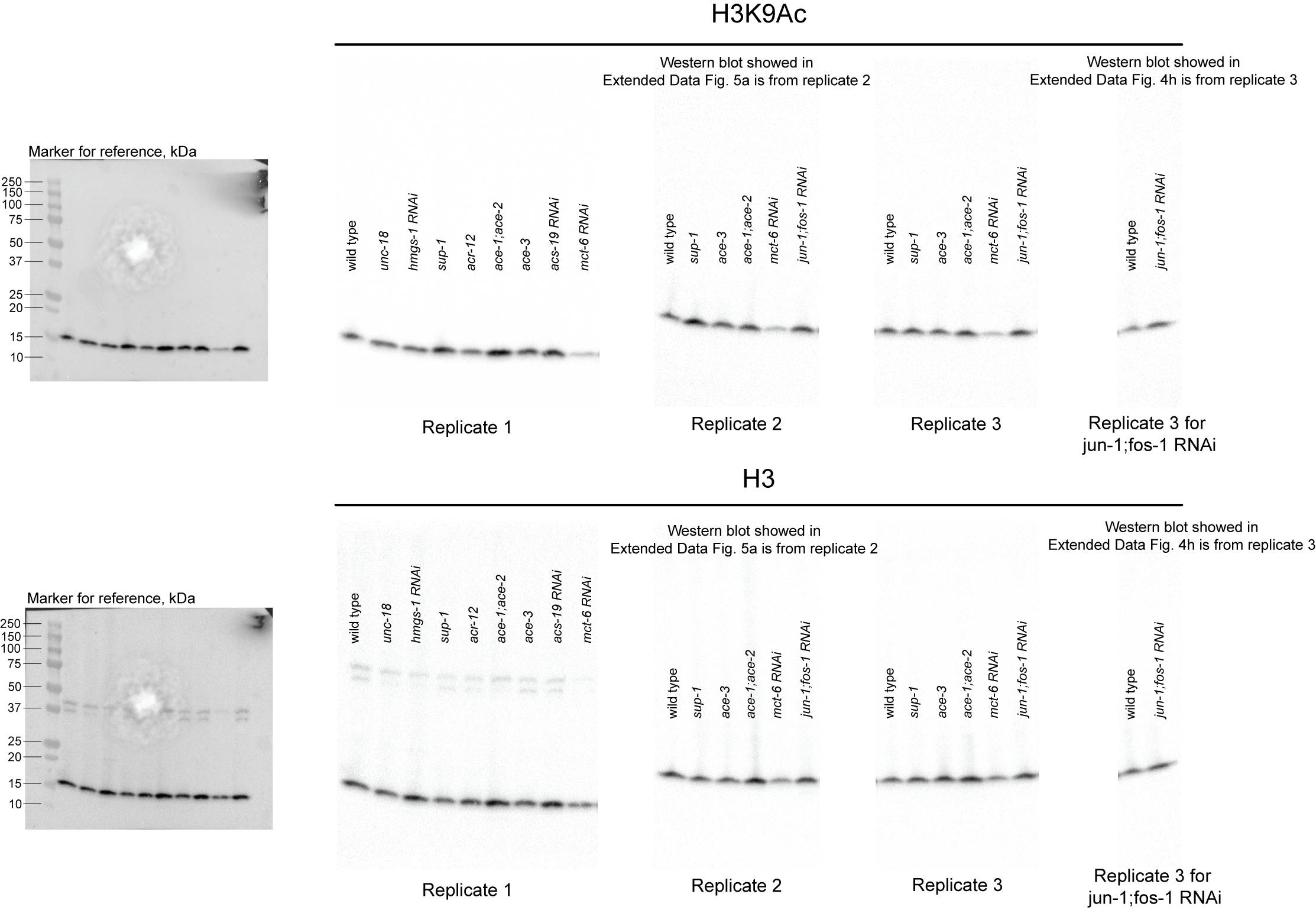

Supplement: Supplementary file 18 — Unprocessed western blots. [file 43587_2023_517_MOESM18_ESM.tif]
